# Eosinophilic gastroenteritis in small intestine in a child in a remote medical centre required surgery^⋆^

**DOI:** 10.1016/j.ijscr.2020.05.029

**Published:** 2020-05-25

**Authors:** Ameer Kakaje, Noha Hedar, Najat Alali Alahmad

**Affiliations:** a Damascus University, Faculty of Medicine, Damascus, Syria; b Department of Gastroenterology, Damascus Hospital, Damascus, Syria; c General Surgery Department, Alhekma Hospital, Aleppo, Syria

**Keywords:** Eosinophilic gastroenteritis, Paediatric, Middle East, Remote area, Public health, Emergency medicine

## Abstract

**Introduction:**

Eosinophilic gastroenteritis (EGE) is an extremely rare disease of unknown aetiology that can occur at any age, but rarely in children. It usually presents with ambiguous abdominal pain and the symptoms can occur for years before diagnosis. It needs a high suspicion and therefore misdiagnosis can occur.

**Presentation of case:**

We present a case of three-and-a-half year old girl who had a complicated EGE but was misdiagnosed as no proper consult was available as she presented in a remote medical centre. She had 50 cm from her small intestine dissected as micro abscesses and a necrotic mass of 3.5*2.5 cm were observed.

**Discussion:**

EGE is usually managed medically. However, the patient’s condition was complicated and surgery was indicated. However, if proper diagnosis was made, conservative surgery would be the proper management as this case tends to improve with steroids.

**Conclusions:**

Medical centres in remote areas should have a way to discuss atypical cases with a skilled consultant even when urgent as surgery indication can completely differ. This is the first case reported in the region with EGE in a child which can justify the difficulty in diagnosis as it is quite rare and have not been reported previously in the region which can lead to futuristic misdiagnosis.

## Highlights

• Eosinophilic gastroenteritis in small intestine in a child which was treated with surgery.
• First study in a child in the Middle East and Levantine.
• Was diagnosed in a remote centre which emphasise the importance of communication between centres.

## 1 Introduction

Eosinophilic gastroenteritis (EGE) is an extremely rare disease that occurs when eosinophilic cells infiltrate the gastrointestinal (GI) tract and can present with many unspecific GI symptoms [1,2]. We present a case of a young girl, aging three years and a half presenting with unspecific abdominal pain which was suspected to be intestinal intussusception and was surgically excised. The lesion was found later to be EGE which made this the first paediatric case to be reported in children in the Levant and the Middle East as only adult EGE was previously reported in the Middle East [3]. It also emphasises the importance of performing imaging when available before surgery and pathology role, even in remote areas and emergent cases as it can spare the patient a possible unnecessary surgery as this patient had 50 cm of her small intestinal resected possibly unnecessarily. This work has been reported in line with SCARE criteria which helped to ensure the high quality of case reports [4].

## 2 Case report

**Fig. 1:**
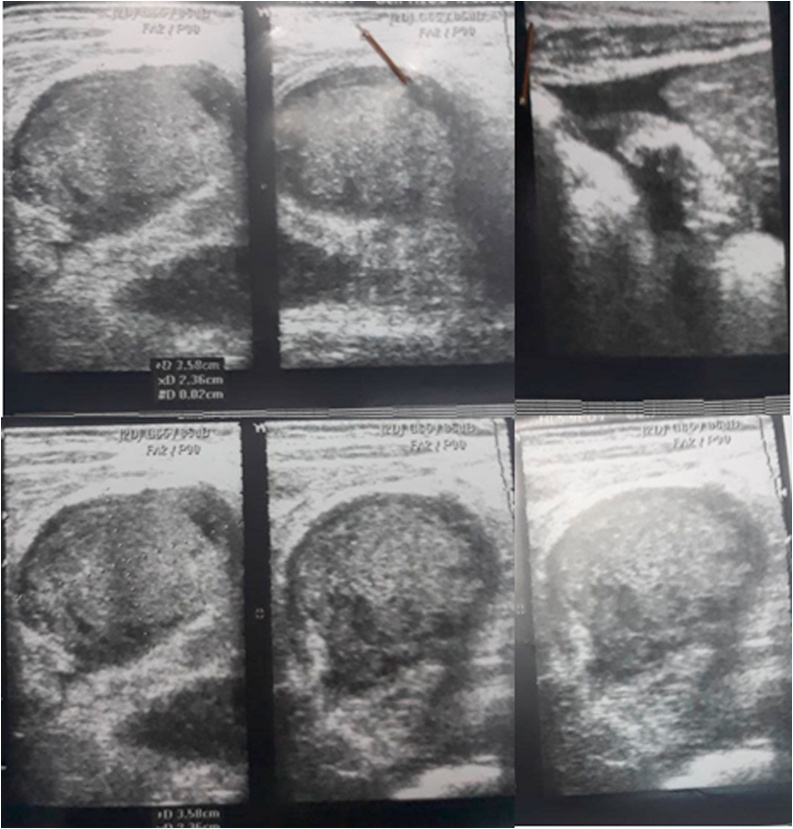
Ultrasonography of the oval mass of 2.5*3.5 cm which was suspected to be acute intussusception.

**Fig. 2:**
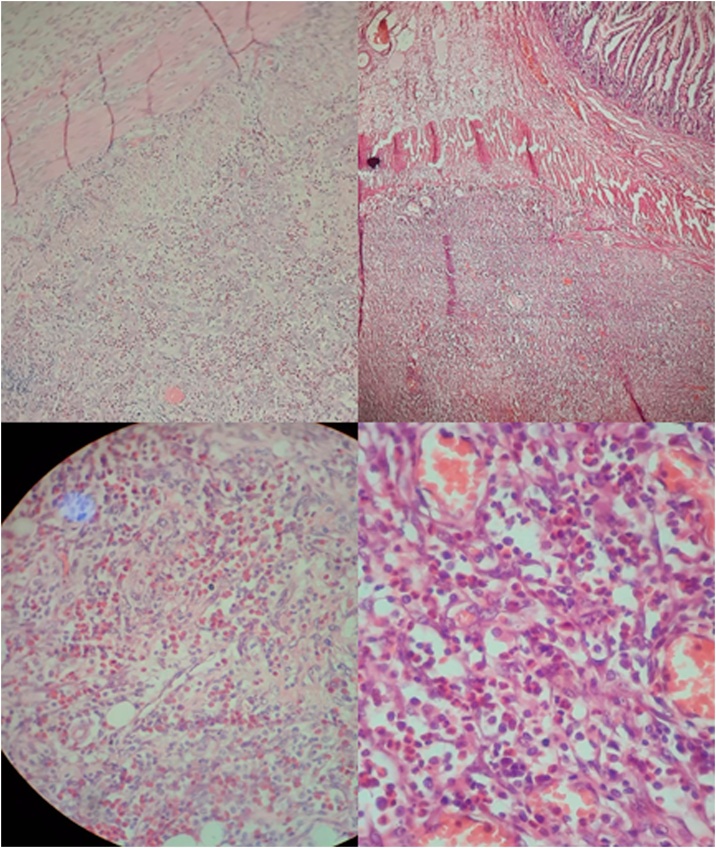
Microscopy of the removed small intestine piece; the upper two photos show the intestine wall with infiltration by eosinophils and the lower two show a more magnified view.

A 42-month-old girl presented to the emergency department as she had recurrent abdominal pain for one week, upper to the umbilicus region. This pain continued for 30 min at a time, but then became continuous. She reported having fever and chills. The pain woke her up at night without any particular position that relieved her pain. The patient had recurrent sour vomiting without any relief. Passing gases and stools did not relief the symptoms and she had no diarrhoea. Past and family history was unremarkable except for asthma in the grandmother. The patient had no food or any other allergies. On examination, the patient weight and height were normal according to her age. She had generalised tenderness over the abdominal area without organomegaly. Bowel movements were normal. Rectal examination showed no stool. Her labs showed a high white cell count of 29,000 * 10^9^ per L (Neutrophils = 77 %, lymphocytes = 17 %, monocytes = 5%, eosinophils = 1%), haemoglobin level of 10.6 g/dL, and CRP of 94.2 mg/L. Platelets count, electrolytes, creatinine, urea, liver function tests, and Widal and Wright tests were all normal. Urine test showed 12–14 white blood cells with + oxalate level. Ultrasonography showed free fluids in the Morison’s pouch in the abdomen and an epileptic mass was observed within the small intestine. It was suspected to be intussusception with a size of 2.5*3.5 superiorly to the umbilicus and to the right (Fig. 1). The rest of abdomen was normal. Doppler ultrasound did not demonstrate blood flow to the mass. The differential diagnosis was intestinal intussusception with necrosis, a complicated appendicitis, intestinal perforation and abdominal abscess. Metronidazole and ceftriaxone were indicated. As the patient was in a rural area, urgent surgery with resection was performed; a 5 cm supra-umbilical incision was made and 50 cm of small intestine was resected, around 100 cm away from the ileocecal junction as the lesion had necrosis and abscesses along with the intestine. Examination of the rest of abdomen was normal. Gross examination reported an excisional biopsy of the ileum with rubbery wall and marked serosal thickening, measuring 30 cm. Microscopic examination (Fig. 2) found purulent inflammation infiltration of the fat in the serosa and sub-serosa with granulomatous tissue and massively eosinophilic inflammatory infiltration (more than 20/hpf). It found 12 isolated reactive lymph nodes. Sections from the separated omental piece revealed fat necrosis with abundant inflammatory granulation tissue. The diagnosis of acute purulent serositis with EGE was made. Patient’s condition was improved with only Cromolyn sodium for the next six months (Fig. 1, Fig. 2).

## 3 Discussion

Pathology for EGE shows infiltration of eosinophils to the bowel wall and more than 20 eosinophils/HPF can be seen in at least one focus. Furthermore, necrotising granulomas, prominent vessels, and vasculitis can be seen and usually without other type of inflammatory cells [5]. The diagnosis can be made by the pathology in addition to not having extra intestinal manifestation or other cause of the eosinophilia and having the GI symptoms [6]. Eosinophilia is not known to be a universal phenomenon [6]. No steroid trial was performed for our patient as the diagnosis of EGE was not suspected. EGE is most commonly presented between the third and fifth decades and it is slightly predominant in males [6]. EGE has unknown aetiology and usually presents with abdominal pain and 80 % of patients may have the symptoms for years. Furthermore, the small intestine is the second most commonly affected organ [2,6]. As EGE is extremely rare, it requires a high degree of clinical suspicion [2] which was not available in our case due to the medical centre being at a remote hospital with no proper imaging and the case presenting acutely which required urgent intervention. Corticosteroids are usually the adequate treatment as they have a 90 % response rate but the duration of treatment is unknown, especially in case of relapse [6]. Surgery should be avoided except in cases of complications and recurrence which can occur despite the surgery [6]. Our patient had a complicated EGE which can justify the surgery. However, conservative surgery could have been conducted instead of 50 cm resection. Our patient had small intestine involvement which was the most affected organ in a study in the Middle East among adults [3]. However, our patient had anaemia and submucosa involvement which were very rare in the same study [3].

In conclusion, EGE is a rare disease which requires a strong suspicion to be diagnosed. However, in our case the atypical imaging made the diagnosis very difficult before surgery which emphasises the importance of imaging before surgery, especially with no experienced health workers to give the proper consultants like in remote areas. EGE should be suspected in acute abdominal pain as surgery can be avoided. This is the first case of paediatric EGE and second overall in the Middle East which makes this case quite unique.

## Declaration of Competing Interest

No conflict of interest to declare.

## Funding

This research did not receive any specific grant from funding agencies in the public, commercial, or not-for-profit sectors.

## Ethical approval

Damascus University deanship ethical approval was taken for this research.

## Consent

Consent for using and publishing data from the patient’s guardian was taken for this case report.

## Guarantor

Ameer Kakaje.

## Provenance and peer review

Not commissioned, externally peer-reviewed.

## CRediT authorship contribution statement

**Ameer Kakaje:** Conceptualization, Formal analysis, Software, Writing - original draft, Writing - review & editing. **Noha Hedar:** Methodology, Visualization, Validation, Investigation, Writing - original draft, Project administration. **Najat Alali Alahmad:** Resources, Supervision, Investigation, Writing - review & editing.
